# The Impact of an Outdoor and Adventure Sports Course on the Wellbeing of Recovering UK Military Personnel: An Exploratory Study

**DOI:** 10.3390/sports7050112

**Published:** 2019-05-15

**Authors:** Mariana Kaiseler, Chris Kay, Jim McKenna

**Affiliations:** Institute for Sport Physical Activity and Leisure 1, Leeds Beckett University, Leeds LS6 3QD, UK; Chris.Kay@leedsbeckett.ac.uk (C.K.); J.McKenna@leedsbeckett.ac.uk (J.M.)

**Keywords:** military personnel, psychological wellbeing, outdoor adventure activities, mental health

## Abstract

UK military personnel have faced increased demands over the last three decades; these have affected their wellbeing and caused multiple physical and mental health problems. Currently, bespoke rehabilitation systems may recommend participation in sports programmes. Although research attention has been drawn to the short-term positive effects of these programmes, their long-term impact on psychological wellbeing is unknown. To address this gap, the current study explored the long-term impact of a sports programme on UK military personnel’s ability to make changes in their day-to-day life through the lens of psychological wellbeing. For this purpose, UK military personnel (n = 97) completed an online survey aiming to provide a quantitative and qualitative picture of their experiences of an outdoor and adventure sports programme, underpinned by the basic psychological needs theory, six months following completion. Findings suggest that 75% of respondents found that the course was useful for facilitating adaptive changes. Content analysis suggests that elements of the course seem to satisfy their basic psychological needs of competence, relatedness and autonomy. Activities initiated six months after the course are mostly aligned with improved psychological wellbeing. Useful theoretical and applied implications are discussed.

## 1. Introduction

UK Military Personnel (UK MP) have faced increased demands since 1991, following the Gulf War, Balkan, Iraq and Afghanistan conflicts. As a consequence, the prevalence and magnitude of their physical and mental health concerns has increased. A recent study [1] concluded that at least 67,515 UK veterans are likely to experience a physical or mental health condition at some point in their life as a result of serving between 2001 and 2014. Their physical health problems include a range of musculoskeletal injuries, traumatic brain injuries, spinal cord injuries, and limb amputations originating from the explosion of explosive devices or from gunshot wounds [2]. Further, the demands of dealing with these severe physical health conditions, combined with the exposure to trauma and difficulty of adapting to civilian life can also lead to increased mental health problems [3,4] and suicide rates [5]. 

These multiple issues combine to make it difficult to reintegrate into civilian life [2,6]. Hence, there is a need to provide effective support programmes that improve participant wellbeing in short —and over longer—time frames. One of the current systems available to support UK MP personnel is through participation in sports programmes [3,7]. The rehabilitative effect of such programmes in supporting the physical and psychological wellbeing of military personnel has received increasing interest from scholars in recent years; short-term impacts seem mostly positive. Accordingly, a systematic review by Caddick and Smith [2] exploring the impact of sport interventions on the physical and psychological wellbeing of military personnel reviewed evidence from 11 studies, and findings highlighted positive psycho-social health and wellbeing outcomes following participation. 

More recently, a systematic review by Greer and Vin-Raviv [8] investigating the impact of outdoor-based traditional recreational programmes (including sport) on veterans diagnosed with Post Traumatic Stress Disorder (PTSD) included 13 studies, and findings suggested positive short-term improvements in mental health measures and wellbeing outcomes. Although these results are promising and highlight the short-term benefits, a further understanding of their impact on the long-term day-to-day life of military personnel is still needed. Indeed, from the 13 studies reviewed [8], only one included paper [9] assessed the participants six months after participating in a programme. Considering that reintegration in a civilian society is a well-known challenge for military personnel [3,6,7], understanding the long-term impact of sports programmes on day-to-day life and wellbeing seems to be a research priority. 

In regard to the study of wellbeing and its definition, consensus is still far from being established. Nevertheless, the experience of flourishing and the possibility to explore opportunities to identify one’s potential [10] have received increased research attention from scholars over centuries [11]. Accordingly, philosophers have proposed multiple views of what constitutes living a good life [12], these include reaching human perfection, or satisfying human needs. Over the years, empirical evidence on wellbeing has increased [13], and a clear distinction has been established between eudaemonic and hedonic wellbeing [14]. Hedonic or subjective wellbeing relates to pleasant and unpleasant life experiences and happiness [15]. Eudaemonic or psychological wellbeing (PWB) refers to the individual’s realisation of their true potential [16], including their experience of purpose and meaning in life [17]. Considering the foundational importance of PWB in defining who an individual is, its adaptive functioning nature [14] and protective features in dealing with significant life challenges and overall health [14,17], it seems wise that military personnel engage in daily activities that foster PWB. Although existing evidence supports the link between participation in physical activity (PA) and positive hedonic wellbeing [18], less is known about the influence of PA participation on eudaemonic wellbeing. According to a recent conceptual framework in the field of adventure recreation [19], participation in activities that support the satisfaction of basic psychological needs have the potential to positively impact eudaemonic wellbeing. Following this line of enquiry, the current study will explore the long-term impact of a sports programme informed by the basic psychological needs theory on UK MP’s ability to make changes in their day-to-day life through the lens of PWB.

## 2. Materials and Methods

### 2.1. Participants

The 97 participants had all attended a 5-day residential Battle Back Multi Activity Course (MAC) in an Outdoor and Adventure (OA) sports context six months previously. Participants were Wounded, Injured or Sick UK MP who had attended a MAC and who met the following inclusion criteria: (i) male and female UK Service personnel; (ii) either wounded (battle field casualties), injured (non-battle casualties) and/or sick (mental/physical illness); and (iii) independently mobile and self-medicating. Out of the 66 participants that provided full answers to all questions at the six months follow-up time point, 18 were female and 48 were male. At the time of attending the MAC, all were serving members of the UK armed forces and were receiving formal recovery support due to being wounded, injured or sick (with a mental health issue or systemic illness). When participants provided information for this current study, they may have been medically discharged from the armed forces, returned to active duty or still be in-service and receiving recovery support. The proportions of participants in each category was not identified as this was not addressed within the ethical approval secured for the on-going evaluation of the within-MAC experiences. All participants, regardless of their subsequent status, had given approval for that activity. Ethical approval was awarded by Leeds Beckett University and The Ministry of Defence Research Ethics Committee (Protocol number: 562 MoDREC 14). A participant information sheet was provided to MAC participants at least 24 h prior to attending. Written informed consent was then obtained from participants upon arrival and continued consent was confirmed online prior to completing the follow-up survey six months after attending. 

### 2.2. MAC Overview

The five-day MAC targets individuals that have already left the Armed Forces and uses adaptive sport and adventurous training to foster personal development and growth. The MAC aims to support participants to achieve their best possible recovery in the transition to civilian life. The MAC combines adaptive sport and adventurous training activities that are relevant to the participants’ recovery plan and are simultaneously enjoyable. Some examples of adaptive sports include indoor climbing and caving, clay pigeon shooting, kayaking and mountain biking. The MAC uses a participant-centred approach, underpinned by the basic psychological needs theory [20]. 

The course provides daily opportunities for participants to develop the three psychological needs of autonomy, competence and relatedness. Particularly, the MAC includes education sessions on the biology of thought processing which are then discussed and applied to the activity sessions. Additionally, opportunities are provided to master sport-based tasks that are important to participants. Furthermore, competence is amplified through daily end-of-day classroom sessions dedicated to reflecting on achievements in knowledge, skills, and mastery of meaningful tasks. The course staff deliberately features military representatives and autonomy is based on a challenge by choice approach, in which participants can refuse to engage in practical activities. To accentuate this experience, and to distinguish the programme from conventional military OA, by design, there are no morning or evening parades, no uniform and no formal recognition of military rank. Finally, the five-day residential course fosters opportunities to promote a strong sense of belonging and connectedness with others in similar situations through sport and social activities, combined with a close relationship with staff, equivalent to around 50 h of contact time.

### 2.3. Measures

Participants completed an online survey, part of a larger research project aiming to assess the quality of the MAC course. For the purpose of the current study, one closed and two open-ended questions were analysed in order to explore UK MP experiences of the MAC course following six months. For this purpose, the closed question asked: “Since being at Battle Back did you make any changes in your day-to-day life?” and participants could select Yes or No. The first open-ended question followed “If yes, what changes have you made? Use the example sentence *I have started* to help…”. The second open-ended question aimed to explore what elements of the course the participants valued the most: “What part of the Multi Activity course had the greatest impact on you?”. 

### 2.4. Data Analyses

Participants’ open-ended responses were analysed using content analyses acknowledging their recognized usefulness for health research [21]. All such responses were coded by the first and second authors independently using an abductive approach, allowing flexibility in the analyses by using pre-identified codes according to Ryff’s model of PWB [17] and the basic psychological needs theory [20], respectively, for open questions one and two, whilst remaining open to the appearance of new themes [22]. Ryffs’s model of PWB proposes six specific dimensions of wellbeing, including self-acceptance (*SA*), the ability to see and accept one’s strengths and weaknesses; purpose in life (PL), refers to having aims and objectives that give life meaning and direction; personal growth (PG), the feeling that personal talents and potential are being realized and developed over time; positive relations with others (PRO), possessing close and valuable connections with significant others; environmental mastery (EM), ability to manage the demands of everyday life and the surrounding world; and autonomy (A), pursuing a sense of self-determination and the strength to follow personal ideas, values and convictions, even if they go against others’ ideas. The basic psychological needs theory [20] is underpinned by the satisfaction of three basic psychological needs for autonomy, competence and relatedness. Autonomy (A) relates to feeling the origin of one’s actions and decisions, and having a sense of control over activities and life. Competence (C) refers to the need of feeling effective in their ongoing interactions with their environment and experiencing opportunities to practice and express their capacities. Relatedness (R) includes the need to belong and connect with others, caring about and being cared for by others, and having a sense of belonging both with other individuals and community. When participants mentioned more than one activity, we coded the first answer named and then the second one, so each response was consistently given one or more codes, as appropriate. The first and second authors coded all questions independently. The first and second coders agreed on 86% of the emerging codes. For the 14% of different codes, further critical discussions established consensus, so all responses were coded.

## 3. Results

### Survey Results

Out of the 97 UK MP that voluntary participated,89 provided responses to the closed and two open-ended questions. In response to the question “Have you made any change since your last sport course at Battle Back?”, 66 (74%) answered *Yes* and 23 (26%) answered *No*. In response to the question “If yes, what changes have you made? Use the example sentence “I have started…” to help.”, out of 66 answers provided, some participants mentioned more than one change, providing a total of 68 responses to be coded. All 68 responses were coded, apart from one that was coded as ‘other’ as it did not fit any of the major codes. Of the 68 coded responses, all were in line with Ryff’s [17] conceptualization of PWB: self-acceptance, purpose in life, personal growth, positive relations with others, environmental mastery, autonomy. Most daily changes reported implicitly suggest activities relating to the more positive dimensions of PWB, with the exception of one answer that suggests limited adaptation to daily life (Table 1 provides an overview of data coded for each PWB dimension). Results suggest that most changes in day-to-day life seem to be beneficially related with PWB. Particularly, daily activities initiated seem to be more prominently related with the environmental mastery, self-acceptance and purpose in life dimensions of PWB. For the second open-ended question “What part of the Multi Activity course had the greatest impact on you?”, out of 66 responses provided, some participants mentioned more than one aspect of the course, providing a total of 79 responses to be coded. The 79 coded responses are alligned with the three basic psychological needs of competence, relatedness and autonomy experienced during participation in the course activities (Table 2 for details). 

## 4. Discussion

This study offers a unique contribution to the OA literature by providing follow-up data of change following a bespoke, theoretically oriented programme aiming to enhance the personal development of wounded, injured and/or sick UK MP. Overall, six months after completion, almost three quarters of MAC participants perceived that they had made valuable changes in day-to-day life. Findings suggest that the MAC helped initiate positive activities likely to foster eudaemonic wellbeing for military personnel. 

As well as being highly adaptative, these changes are related with all dimensions of psychological wellbeing [23] and the relevant aspects of the course reported are aligned with the satisfaction of the three basic psychological needs of autonomy, competence and relatedness [20]. The most prominent change initiated by participants was linked to environmental mastery, notably (i) being more active on a daily basis, (ii) initiating sport participation, and (iii) improving multiple health behaviours (e.g., diet and hydration). These findings show the positive impact of the MAC on near-transfer, which is to say, the effect on activities similar to that of the MAC but in different contexts. When explaining why this particular aspect of psychological wellbeing featured so strongly over others, it is likely to reflect the satisfaction of the psychological need of competence developed by the OA course programme. As stated by participants, the course “helped me to recognise the positive qualities and skills I have”, suggesting that the programme generated powerful experiences, enhancing the ability to stay active and manage health behaviours.

Our findings also support the long-term impact of OA programmes for UK MP. Indeed, these seem to benefit the initiation of activities related with a higher sense of self-acceptance and purpose in life, personal growth, autonomy and relations with others. Possible explanations for the findings are likely to be aligned with a combination of factors underpinning OA sport participation and personal growth, as well as course design informed by a participant-centred approach and basic psychological needs theory. In support of the first explanation, a recent review on the benefits of OA sports to society [24] supports the short-term effects of OA sports programmes on physical and mental health outcomes and wellbeing. Accordingly, our findings extend this knowledge to show the positive long-term benefits particularly among military personnel. With regard to psychological wellbeing, evidence [25] highlights the potential of participant-centred interventions to improve positive self-care behaviours and health outcomes. 

The positive long-term outcomes of the OA sports programmes are promising original insights. With regard to psychological wellbeing, while the evidence suggests lower levels in those with diverse physical and mental impairment, it is also important to acknowledge the scale of (i) individual gains and/or (ii) the maintenance of wellbeing following impairment [14]. Importantly, evidence increasingly documents the wider implications of psychological wellbeing in health, biological regulation and brain functioning; the common underlying theme is that the MAC appears to facilitate adaptive and protective purposes by satisfying needs of relatedness, competence and autonomy. 

Considering the well-established importance of the eudaemonic concept of wellbeing in other areas of psychology and its theoretical relevance in supporting wounded military personnel transitioning from military to civilian life, this paper advocates the need to integrate the basic psychological needs theory in the design of military personnel future rehabilitation sport programmes. Additionally, the evaluation component should not only consider short-term but also long-term effects of OA programmes on psychological wellbeing. Additionally, acknowledging that most research conducted in this area [8] mainly considers narrow impacts on negative outcomes (e.g., PTSD symptomology), addressing a wider perspective on positive outcomes (i.e., flourishing and positive functioning) assures that UK MP are supported in their reintegration post the course.

Though this study provides a novel insight into the long-term benefits of OA sports programmes in improving UK MP psychological wellbeing, it is important to consider the findings in light of the study’s limitations and consider recommendations for future research. First, the study was exploratory in nature and relied on survey data only, restricting the depth of information collected. Future research should consider using more comprehensive qualitative methods (e.g., interviews, daily diaries) to fully understand UK MP’s long-term experiences of psychological wellbeing following OA sports programmes. In addition, considering that data was collected six months after the course, we recommend future research to include measurements at more regular interval points, for example straight after the course and following six months, to fully understand the long-term sustainability of OA sports programmes’ changes on UK MP’s psychological wellbeing. Furthermore, acknowledging recent advances within the basic needs framework to consider beneficence as a fourth basic psychological need, showing to influence eudaemonic wellbeing [26], it is recommended that future research explores the utility of this variable in explaining psychological wellbeing for military personnel during and following OA programmes. Finally, acknowledging previous literature highlighting the important role of nature in enhancing psychological wellbeing [27], it is recommended that future studies explore the importance of this variable in OA programmes for military personnel.

In conclusion, the current study offers an original and significant addition to the literature by showcasing the long-term positive impact of OA sports programmes in supporting UK MP’s ability to make positive changes in their day-to-day life. Furthermore, the work provides useful theoretical insights on the use of the basic psychological needs theory to inform the design of OA sports programmes aiming to increase eudaimonic approaches to psychological wellbeing [14], facilitating post-MAC recovery. 

## Figures and Tables

**Table 1:** Main themes for the question “have you made any changes since your last sport course at Battle Back? If yes, what changes have you made? I have started…”. (n = 66).

Theme	No Change Example Quotes	Change Example Quotes	Frequency n (%)
Self-Acceptance (SA)	–	“Thinking about a positive thought for each day.”	16 (24%)
Positive Relations with others (PRO)	–	“Tolerating other people’s faults more readily.”	5 (7%)
Autonomy (A)	–	“Facing my issues myself and not expecting support or help...”	5 (7%)
Environmental Mastery (EM)	–	“Adaptive swimming and cycling on a very regular basis.”	23 (35%)
Purpose in Life (PL)	“I have tried cheer myself up/be happy but still can’t find any happiness in myself or life.”	“Have started work as a teacher/lecturer.”“Going back to work.”	12 (18%)
Personal Growth (PG)	–	“I have moved abroad to start my new life.”	7 (10%)

*Note:* responses could be coded as more than one theme.

**Table 2:** Main themes for the question “What part of the Multi Activity course had the greatest impact on you?” (n = 66).

Theme	Elements of the Course Example Quotes	Frequency n (%)
Autonomy (A)	“Blend of activities particularly those related to life. I remember the mountain biking, for example, look at where you want to go, not at the hazard-brilliant!” “I can review what I can control and I can’t control…”	14 (19%)
Competence (C)	“…I had feared after my illness I would be physically diminished- Battle Back helped me to kick that fear into the long grass.”“The course made me feel like the old me.” “The course helped me to recognize the positive skills and qualities I have.”	38 (57%)
Relatedness (R)	“The camaraderie and the realization that I am not alone feeling as I do.” “…being able to interact with people who are in similar situation and being able to speak to staff who are happy to help.””	27 (40%)

*Note:* responses could be coded as more than one theme.
