# Correction: Kaiseler, M., et al. The Impact of an Outdoor and Adventure Sports Course on the Wellbeing of Recovering UK Military Personnel: An Exploratory Study. *Sports* 2019, *7(5)*, 112

**DOI:** 10.3390/sports8090122

**Published:** 2020-09-03

**Authors:** Mariana Kaiseler, Chris Kay, Jim McKenna

**Affiliations:** Institute for Sport Physical Activity and Leisure 1, Leeds Beckett University, Leeds LS6 3QD, UK; Chris.Kay@leedsbeckett.ac.uk (C.K.); J.McKenna@leedsbeckett.ac.uk (J.M.)

The authors wish to make the following corrections to this paper [1]:

On page 3, Section 2.2, Paragraph 1, “The five-day MAC targets individuals that have already left the Armed Forces and uses adaptive sport and adventurous training to foster personal development and growth.” should read “The five-day MAC targets individuals that have **not** already left the Armed Forces and uses adaptive sport and adventurous training to foster personal development and growth.”

The authors would like to apologize for any inconvenience caused to the readers by these changes.
